# eHealth Literacy and Web 2.0 Health Information Seeking Behaviors Among Baby Boomers and Older Adults

**DOI:** 10.2196/jmir.3992

**Published:** 2015-03-17

**Authors:** Bethany Tennant, Michael Stellefson, Virginia Dodd, Beth Chaney, Don Chaney, Samantha Paige, Julia Alber

**Affiliations:** ^1^ICF International9300 Lee HighwayFairfax, VAUnited States; ^2^Center for Digital Health and WellnessDepartment of Health Education and BehaviorUniversity of FloridaGainesville, FLUnited States; ^3^Department of Community Dentistry and Behavioral ScienceUniversity of FloridaGainesville, FLUnited States; ^4^Department of Health Education & PromotionEast Carolina UniversityGreenville, NCUnited States

**Keywords:** social media, aging, health literacy, Web 2.0

## Abstract

**Background:**

Baby boomers and older adults, a subset of the population at high risk for chronic disease, social isolation, and poor health outcomes, are increasingly utilizing the Internet and social media (Web 2.0) to locate and evaluate health information. However, among these older populations, little is known about what factors influence their eHealth literacy and use of Web 2.0 for health information.

**Objective:**

The intent of the study was to explore the extent to which sociodemographic, social determinants, and electronic device use influences eHealth literacy and use of Web 2.0 for health information among baby boomers and older adults.

**Methods:**

A random sample of baby boomers and older adults (n=283, mean 67.46 years, SD 9.98) participated in a cross-sectional, telephone survey that included the eHealth literacy scale (eHEALS) and items from the Health Information National Trends Survey (HINTS) assessing electronic device use and use of Web 2.0 for health information. An independent samples *t* test compared eHealth literacy among users and non-users of Web 2.0 for health information. Multiple linear and logistic regression analyses were conducted to determine associations between sociodemographic, social determinants, and electronic device use on self-reported eHealth literacy and use of Web 2.0 for seeking and sharing health information.

**Results:**

Almost 90% of older Web 2.0 users (90/101, 89.1%) reported using popular Web 2.0 websites, such as Facebook and Twitter, to find and share health information. Respondents reporting use of Web 2.0 reported greater eHealth literacy (mean 30.38, SD 5.45, n=101) than those who did not use Web 2.0 (mean 28.31, SD 5.79, n=182), *t*_217.60_=−2.98, *P*=.003. Younger age (*b*=−0.10), more education (*b*=0.48), and use of more electronic devices (*b*=1.26) were significantly associated with greater eHealth literacy (*R* ^2^ =.17, *R* ^2^adj =.14, F_9,229_=5.277, *P*<.001). Women were nearly three times more likely than men to use Web 2.0 for health information (OR 2.63, Wald= 8.09, df=1, *P*=.004). Finally, more education predicted greater use of Web 2.0 for health information, with college graduates (OR 2.57, Wald= 3.86, df =1, *P*=.049) and post graduates (OR 7.105, Wald= 4.278, df=1, *P*=.04) nearly 2 to 7 times more likely than non-high school graduates to use Web 2.0 for health information.

**Conclusions:**

Being younger and possessing more education was associated with greater eHealth literacy among baby boomers and older adults. Females and those highly educated, particularly at the post graduate level, reported greater use of Web 2.0 for health information. More in-depth surveys and interviews among more diverse groups of baby boomers and older adult populations will likely yield a better understanding regarding how current Web-based health information seeking and sharing behaviors influence health-related decision making.

## Introduction

Over the past several decades, inequities in Internet availability and accessibility have diminished due to technological advances and lower-cost access to broadband Internet. Currently, over 2.8 billion people use the Internet worldwide [1], with estimates indicating that nearly 90% of adults regularly access the Internet for information [2]. Greater access to the Internet has increased the availability of health information [3-5], yet many Internet users continue to face challenges accessing relevant and literacy-sensitive health and medical content that is of high quality [4,6-13]. Individuals without adequate skills to navigate the Internet may also unknowingly access health information that is inaccurate and potentially dangerous to their overall health [11,12,14]. This phenomenon is especially problematic for the aging population who is at particularly high risk for disability and chronic disease [15]. Compared to their younger counterparts, older adults are more likely to have lower health literacy that negatively impacts health care access, chronic disease management, and health status [16,17].

Although older adults are traditionally “late adopters” of technology, research conducted by the Pew Research Center’s Internet and American Life Project indicates that more than half (59%) of adults 65 years and over [18], and 88% of baby boomers between 50 and 64 years access the Internet [19]. Approximately 74% of older adults and 88% of baby boomers use a cellular device, and an increasing number are now beginning to use advanced digital devices with mobile Internet access [20]. One common reason that baby boomers and older adults use these electronic devices is to seek out relevant Web-based health information [21]. For example, in a recent study by Medlock et al [22], researchers found that the Internet was a trusted source of health information among older adults, especially for learning more about the prognosis, symptoms, and treatment options for personal health issues.

**Figure 1:**
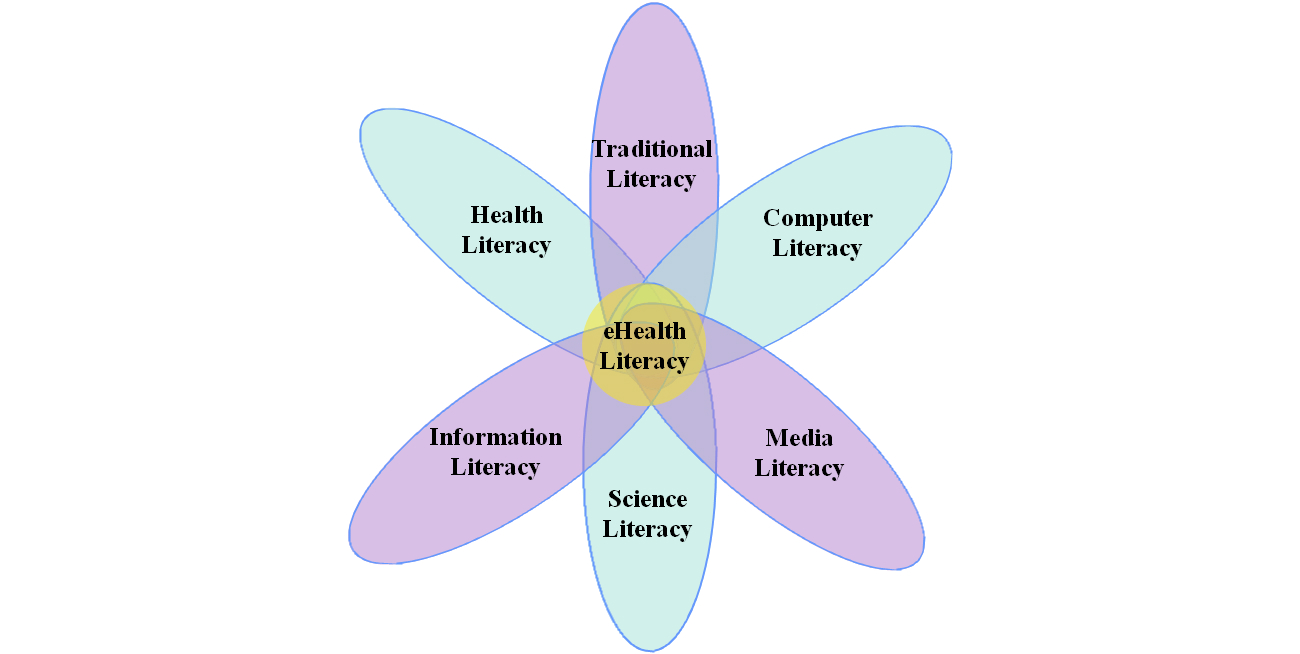
eHealth literacy Lily Model [25].

While the older adult population is becoming more and more reliant on the Internet to locate and obtain health-related information and services [2,6,23], baby boomers and older may struggle to possess adequate eHealth literacy [12,24]. eHealth literacy is defined as the ability to seek, find, understand, and appraise health information from electronic resources and apply that knowledge to solving a health problem or making a health-related decision [25]. The construct of eHealth literacy represents a foundational skill set that combines six forms of literacy that extend beyond traditional definitions of health literacy and numeracy to include: (1) traditional, (2) information, (3) media, (4) health, (5) scientific, and (6) computer (Figure 1) [25].

Two context-specific domains of eHealth literacy that are particularly noteworthy to measure among Internet users beyond the age of 50 years include *health* and *computer* (or *digital*) literacy [24,26,27]. Health literacy is defined as the degree to which individuals can obtain, process, and understand the basic health information and services they need to make appropriate health decisions [28]. Results from a systematic literature review that examined the role that health literacy plays in the treatment of older adults indicated that lower health literacy is associated with increased health care costs, more medication errors, ineffective and undesirable patient-provider communication, as well as inefficient use of health care services [29]. Computer (or digital) literacy involves an individual’s ability to adapt to new technologies productively and efficiently to solve problems or answer questions through the operation of an electronic device [25,26]. Computer literacy is especially important to consider among the aging population because adroit use of technology may help reduce cognitive decline among older adults 50 to 89 years [30]. Research suggests that determinants of computer literacy include knowledge about technology [21], exposure to electronic devices [31], and the type and number of electronic devices that are used [25]. Low computer literacy in older adults often precludes these populations from successfully accessing and deciphering high-quality sources of Web-based health information [6,26,32]. Both health and computer literacy are not static; rather, they are influenced by an individual’s health status, motivation, education level, and changes in technology [25]. Without adequate attention to health and computer (or digital) literacy among the aging population, there is a risk of reopening the digital divide, solidifying current health disparities, and perpetuating inequities that result in behavioral risk factors that compromise patient safety and reduce health outcomes among these vulnerable populations.

While the Internet has traditionally been used as a one-way health communication channel (ie, Web 1.0) [33], the concept of “participative Internet” (ie, Web 2.0) has risen in popularity due to the advent of social networking, which facilitates multi-way conversations about health [24,34-36]. Web 2.0 has transformed health communication patterns, allowing users to add information or content on the Web [37] and collaborate with others on issues related to health care [34-36]. Although baby boomers and older adults have traditionally been identified as “passive consumers” of health information on using Web 1.0 [38], Web 2.0 provides new opportunities for promoting health and preventing behavioral risk factors associated with chronic disease. A recent study suggests that adults between the ages of 50 and 60 years living with a compromised health status utilize the Internet for health care purposes because they want to be active in their health care decision-making [39]. For example, some older adults use email and interactive communication tools on the Internet to promote cancer screening to their peers [40]. Virtual discussion-based forums for patient engagement now also target individuals living with long-term health problems [31,35,41,42], which has caused the number of customized Internet applications for chronic disease-related behavioral risk management to grow [42,43]. Individuals with a primary health care provider, chronic disease, and those who are younger are more likely to use social networking sites for health-related activities [44]. However, baby boomers and older adults report not accessing or utilizing Web 1.0 and Web 2.0 for a number of reasons, including the high cost of devices and Internet access, insufficient knowledge about device function, and poor perceived self-efficacy [31,45].

Currently, there is a dearth of information regarding which sociodemographic and social determinant variables, other than age, education, and income [38], are associated with eHealth literacy and use of Web 2.0 for health care purposes among baby boomers and older adult populations. Preliminary research suggests that eHealth literacy is negatively associated with age among low income homebound adults above 60 years of age [31], but the literature is not definitive regarding relationships between social determinants, sociodemographic variables, eHealth literacy, and use of social networking sites for health promotion [36,37]. Some research indicates that baby boomers are significantly more likely than older adults to use health information websites, email, automated call centers, medical video conferencing, texting, and podcasts for health care purposes [46], but it is unclear whether aging populations have confidence in their ability to utilize these technologies to find and evaluate Web-based health information. The Structural Influence Model of Health Communication (SIMHC) postulates that different forms of media and different genres within a medium (ie, using the Internet as a one-way communication channel, Web 1.0 vs using social media as a two-way communication channel, Web 2.0) may differentially influence health information seeking and sharing behaviors among different populations [47]. Further, SIMHC posits that media communications influences health by raising awareness, focusing attention on health, highlighting relevant health issues, providing health information, and reinforcing health-related knowledge, attitudes, and behaviors [48].

**Figure 2:**
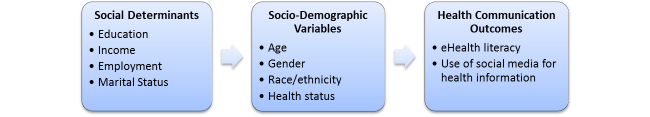
Adapted Structural Influence Model of Health Communication.

The demographic composition of the United States will undoubtedly be reshaped by the baby boomer generation in the coming decades. Baby boomers already make up a large proportion of the population [49], and by 2029, 20% of the United States population will be over the age of 65 years [49]. With increased age comes concomitant demands for health care resources; therefore, it is important to examine whether people in the baby boomer and older adult age group are confident in their ability to access and effectively navigate Web-based health resources to obtain quality health information that will allow for informed decision making. At the current time, it is unknown whether or not health status or electronic device use is associated with eHealth literacy and/or use of Web 2.0 for health promotion among adults 50 years and older [50]. Therefore, the purpose of this study was to determine the extent to which social determinants such as electronic device use and sociodemographic variables included in the SIMHC (Figure 2) were associated with distinct health communication outcomes (ie, eHealth literacy and use of Web 2.0 to find and evaluate health information), in baby boomers and older adults.

## Methods

### Recruitment

In February 2013, a cross-sectional telephone survey was conducted as part of the state of Florida Consumer Confidence Index (F-CCI) Survey, administered by the University of Florida Bureau of Economic and Business Research (BEBR). The BEBR conducts and disseminates demographic and economic research on residents of the State of Florida to inform public policy [51]. At least 500 households in the state are surveyed on a monthly basis, using the random digit dialing (RDD) method. A minimum of 10 call attempts are placed per household. Dillman supports the use of telephone surveys for collecting data among aging populations who often feel reassured that they are speaking with an actual person on the telephone, as opposed to answering questions via other modes (eg, paper-based, Internet) [52]. Throughout February 2013, telephone surveys were administered Monday through Friday between 9 AM and 9 PM, Saturdays between 12 PM and 6 PM, and Sundays between 3 PM and 9 PM. A total of 6695 telephone calls were placed during this time period, and a total of 493 individuals agreed to complete the telephone survey.

### Participants

Respondents were included in main analyses if they (1) reported being 50 years of age or older, and (2) had ever accessed the Internet or sent/received email messages. The youngest baby boomers just recently turned 50 years of age , having been born between 1946 and 1964 [49]. A total of 393 respondents in the sample reported being 50 years of age or over, yet 110 respondents responded “no” to the following question adopted from the Health Information National Trends Survey (HINTS) [53]: “Do you ever go online to access the Internet or World Wide Web, or to send and receive email?” Therefore, data from a total of 283 respondents was analyzed in this study. Human subjects approval was secured from the university’s Institutional Review Board (IRB) prior to administering the telephone survey or analyzing any participant data.

### Measurement

#### Electronic Device Use

Electronic device use was measured using one item adapted from the HINTS survey [53]. Participants were asked, “In the past 12 months, have you used the Internet on any of the following devices to look for health or medical information for yourself?” Respondents could select any devices from the following list: (1) desktop computer, (2) laptop computer, (3) cell phone, or (4) mobile handheld device like an e-reader or tablet.

#### eHealth Literacy

eHealth literacy was measured using the eHealth Literacy Scale (eHEALS) [54]. The eHEALS determines consumers’ combined knowledge, confidence, and perceived skills finding, evaluating, and applying electronic health information to health problems [54]. The measure consists of 8-items scored on a 5-point Likert scale ranging from 1 (strongly disagree) to 5 (strongly agree). Higher scores on the eHEALS indicates higher eHealth literacy (total score range=5-40). The internal consistency of the data collected using the eHEALS in this study was high (Cronbach alpha=.90), and comparable to reliability estimates reported in previous studies [54,55].

#### Use of Web 2.0 for Health Information

Use of social media (Web 2.0) for health information was measured using one item adapted from the HINTS survey [53]. Participants were asked, “In last 12 months, have you used the Internet for any of the following reasons to locate or share health information?” Respondents could select all reasons for using the Internet: (1) participated in a Web-based-support group, (2) used a social networking site like Facebook/Twitter/ LinkedIn, or (3) wrote in a Web-based diary or blog.

#### Sociodemographic and Social Determinant Variables

Sex (male or female), age (in years), race (Caucasian/white, non-Caucasian/white), ethnicity (Hispanic/non-Hispanic), education (less than high school, high school/GED, some college, college graduate, post-graduate), income (US$) (less than $20,000, $20,000-$49,999, $50,000-$99,999, $100,000 or more), and marital status (married, separated, divorced, widowed, never been married) were all assessed. Perceived health status was also measured using the following scale: (1) poor, (2) fair, (3) good, (4) very good, and (5) excellent.

### Statistical Analysis

SPSS version 21.0 was used to compute frequency and descriptive statistics to summarize sociodemographic and social determinant characteristics, frequency statistics for each eHEALS item, and the number of respondents reporting use of Web 2.0 for health information. An independent samples *t* test was performed to compare eHealth literacy among users and non-users of Web 2.0 for health information. Given that specific technologies and tools must be considered when attempting to examine the use of Web 2.0 in health promotion [56], we also examined whether use of discrete Web 2.0 tools (ie, social networking websites, Web-based support groups, blogs) was associated with eHealth literacy. A multiple linear regression was also conducted to determine whether use of multiple electronic devices (number of digital devices used), sociodemographic variables (sex, age, income, race, ethnicity, education, marital status), and perceived health status as a social determinant predicted overall eHEALS scores. Finally, a multiple logistic regression was conducted to determine whether these predictor variables were associated with the use/non-use of Web 2.0 for health information. Use of Web 2.0 for health information was dummy coded as “0” for participants who had never used social media for seeking or sharing health information and “1” for participants who had reported use of social media for seeking or sharing health information. Analyses were considered statistically significant at the *P*<.05 alpha level (two-tailed).

## Results

### Participant Characteristics

Table 1 describes the characteristics of study participants reporting use of the Internet (n=283). Respondents ranged in age from 50 to 91 years (mean 67.46 years, SD 9.98). Slightly over half of the respondents were male (155/283, 54.8%), and the vast majority identified their race as Caucasian/white (252/283, 89.0%). A small proportion of respondents (16/283, 5.7%) identified as Hispanic. The majority of respondents (186/283, 65.7%) reported being married, yet 15.9% (45/283) were widowed, 12.7% (36/283) were divorced or separated, and 3.9% (11/283) reported never being married. Over 90% of participants (263/283, 92.9%) reported completing high school and over three-quarters attended college (215/283, 75.9%). The largest number of respondents fell into the $20,000 to $49,999 annual income bracket (82/283, 29.0%), followed by $50,000 to $99,999 (80/283, 28.3%), and ≥$100,000 (58/283, 20.5%). Over 50% of participants (165/283, 58.3%) reported “very good” or “excellent” health status.

### Electronic Device Use for Accessing Web-Based Health Information

A little over half of the respondents accessed the Internet through a desktop computer to search for Web-based health information (143/283, 50.5%), and over 40% reported use of a laptop computer (120/283, 42.4%). More than 20% of respondents (58/283, 20.5%) reported using a mobile phone, and 14.5% (41/283) reported use of a tablet computer. Less than half of the respondents (124/283, 43.8%) reported using one electronic device to search for health information, and 30.4% (86/283) reported use of two or more devices.

### Use of Social Media (Web 2.0) for Health Information

Table 2 describes use of Web 2.0 for health information among respondents. Over one-third of respondents (35.7%, 101/283) reported using Web 2.0 to locate or share health information over the past 12 months. However, almost 90% of Web 2.0 users (90/101, 89.1%) reported using only one type of social media for this purpose. Most Web 2.0 users (96/101, 95.0%) reported using popular social media sites such as Facebook and Twitter. Far fewer reported belonging to Web-based support groups (11/101, 10.9%) or contributing to Web-based health diaries/blogs (6/101, 5.9%).

### eHealth Literacy

**Figure 3:**
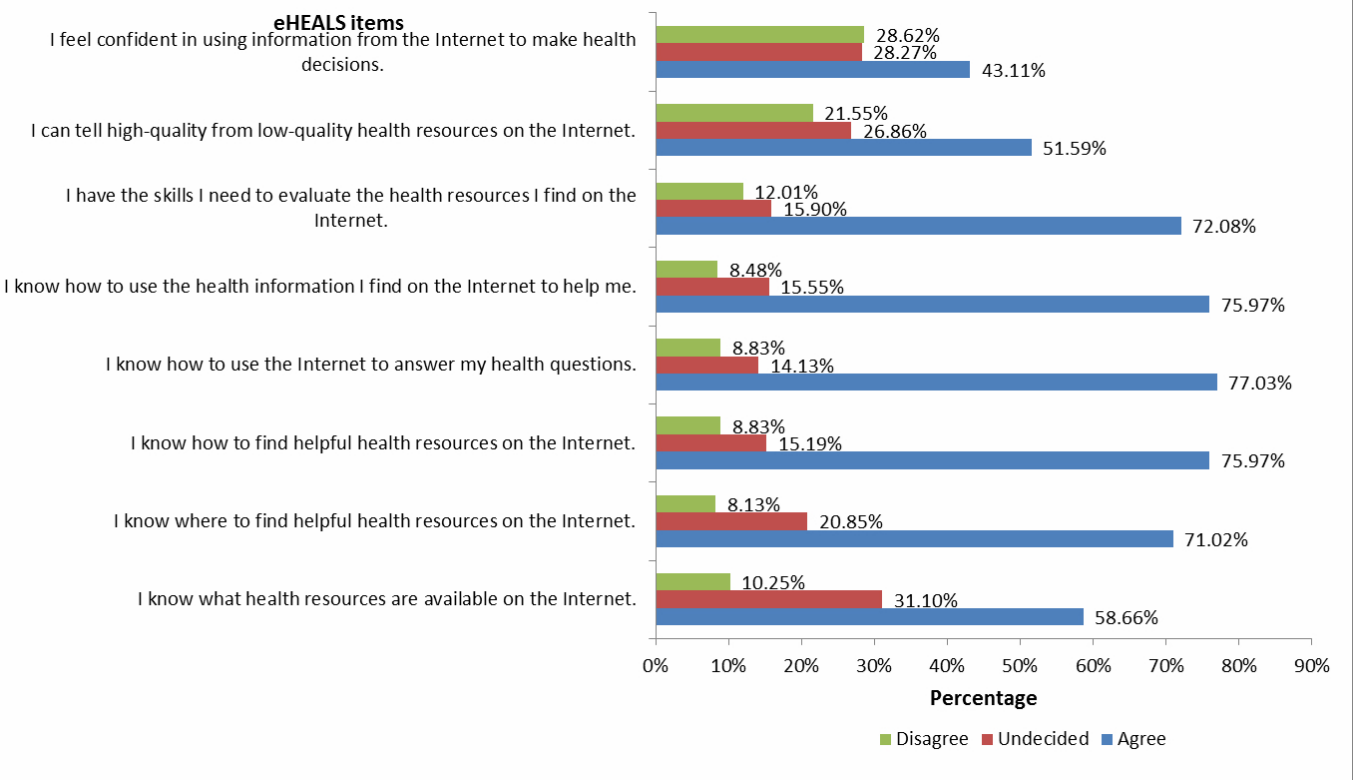
Frequency of responses to 8-item eHEALS (n=283).

Total scores on the eHEALS ranged from 11 to 40 (mean 29.05, SD 5.75). Figure 3 illustrates the response frequencies for each eHEALS item. Over 70% of respondents agreed with the following five statements on the eHEALS: “I have the skills I need to evaluate the health resources I find on the Internet” (204/283, 72.1%); “I know how to use the health information I find on the Internet to help me” (215/283, 76.0%); “I know how to use the Internet to answer my health questions” (218/283, 77.0%); “I know how to find helpful resources on the Internet” (215/283, 76.0%); and “I know where to find helpful health resources on the Internet” (201/283, 71.0%). Two statements with the greatest level of disagreement were related to confidence using Web-based health information to make health decisions (81/283, 28.6%) and the ability to distinguish between high- and low-quality health resources on the Internet (61/283, 21.6%).

**Table 1:** Sociodemographic and health status characteristics of study participants (n*=*283).

Demographics		n (%)
**Sex**		
	Female	128 (45.2)
	Male	155 (54.8)
**Marital status**		
	Married	186 (65.7)
	Widowed	45 (15.9)
	Never married	11 (3.9)
	Divorced or separated	36 (12.7)
	No response	5 (1.8)
**Ethnicity**		
	Yes, Spanish or Hispanic	16 (5.7)
	No, Spanish or Hispanic	264 (93.3)
	No response	3 (1.1)
**Race**		
	White	252 (89.0)
	Black	10 (3.5)
	Asian or Pacific Islander	1 (0.4)
	American Indian or Alaska native	3 (1.1)
	Other	6 (2.1)
	Multi-racial or mixed race	7 (2.5)
	No response	4 (1.4)
**Education**		
	Less than high school graduate	19 (6.7)
	High school graduate/GED	48 (17.0)
	Some college/associates degree	82 (29.0)
	College graduate	70 (24.7)
	Postgraduate	63 (22.2)
	No response	1 (0.4)
**Income (US$)**		
	Less than $19,999	30 (10.6)
	$20,000 to $49,999	82 (29.0)
	$50,000 to $99,999	80 (28.3)
	More than $100,000	58 (20.5)
**Health status**		
	Excellent	62 (21.9)
	Very good	103 (36.4)
	Good	71 (25.1)
	Fair	30 (10.6)
	Poor	14 (4.9)
	No response	3 (1.1)

**Table 2:** Frequency and percentage of baby boomers and older adults who used Web 2.0 to locate or share health information (n=283).

In last 12 months, have you used the Internet for any of the following reasons to locate or share health information?		n (%)
**Popular social media**		
	No	187 (66.1)
	Yes	96 (33.9)
**Web-based support group**		
	No	272 (96.1)
	Yes	11 (3.9)
**Blogs**		
	No	277 (97.9)
	Yes	6 (2.1)
**Report using at least one of these types of social media**		
	No	182 (64.3)
	Yes	101 (35.7)

### Relationship Between Use of Social Media (Web 2.0) for Health Information and eHealth Literacy

There was a statistically significant difference in total eHEALS scores among users (mean 30.38, SD 5.45, n=101) and non-users (mean 28.31, SD 5.79, n=182) of Web 2.0 for health information, *t*_217.60_=−2.98, *P*=.003. Respondents reporting use of Web 2.0 reported greater eHealth literacy than those who did not use Web 2.0. Users of popular social networking sites such as Facebook, Twitter, and LinkedIn for health information, had greater eHealth literacy (mean 30.22, SD 5.49, n=96) than non-users (mean 28.45, SD 5.80, n=187) for health information, *t*_201.28_=−2.20, *P*=.01. Similar to users of popular social networking sites, respondents who reported prior use of Web-based support groups for health-related purposes reported greater eHealth literacy (mean 31.82, SD 3.06, n=11) than those reporting no such involvement (mean 28.94, SD 5.81, n=272), *t*_13.12_=−2.91, *P*=.01. However, there was no statistically significant difference between users and non-users of Web-based diaries/blogs for health-related purposes, *t*_5.40_=−1.80, *P*=.13.

### Predictors of eHealth Literacy

Prior to conducting the multiple linear regression analysis to determine whether sociodemographic, health status, and electronic device use were associated with eHealth literacy, data were examined for multicollinearity. Both the variance inflation factors (VIF) (≤1.48) and tolerance statistics (≤0.89) met recommended cut-off points of less than 10 and greater than 0.10 respectively [57]. These results indicated that the regression model was not adversely compromised. Overall, the model accounted for 18.2% of the variance in eHEALS scores, which was statistically significant, *R* ^*2*^=.18, *R* ^*2*^_adj_ =.14, *F*_9,229_=5.28, *P*<.001. Table 3 presents a summary of the regression coefficients generated by the analysis. Statistically significant predictors of eHealth literacy included age, education, and total number of electronic devices used to seek out health information. As age (*b*=−0.10) increased by 1 year, total eHEALS score decreased by .10 points. This indicated that, on average, the youngest baby boomers of age 50 years were likely to score approximately 1.56 points higher on the eHEALS scale than older adults who were 65 years of age. In addition, as education level (*b*=0.48) increased, total eHEALS scores increased by .48 points. Finally, holding all other factors in the regression model constant, the use of more electronic devices to access Web-based health information (*b*=1.26) was significantly associated with greater eHealth literacy. Sex, marital status, race, ethnicity, income, and health status were not significantly associated with eHealth literacy.

**Table 3:** Multiple linear regression predicting eHealth literacy (eHEALS).

Model^a^	*B*	*SE B*	β
Constant	26.74	3.90^b^	
Sex	1.07	0.73	.10
Age	−0.10	0.04	−.19^b^
Marital status	−0.26	0.37	−.05
Ethnicity	0.32	1.40	.01
Race	0.04	0.35	.01
Education level	0.48	0.18	.18^b^
Income	0.23	0.50	.03
Health status	0.02	0.32	.01
Total number of electronic devices used to seek health information^c^	1.26	0.31	.25^b^

^a^Model *R* ^*2*^=.18, *R* ^*2*^_adj_ =.14.

^b^ *P*<.01, two-tailed.

^c^Participants were asked to report whether or not they used the following electronic devices to seek out health information: desktop, laptop, cell phone, or mobile tablet.

### Predictors of Web 2.0 Use for Health Information

Prior to conducting the multiple logistic regression analysis to determine whether sociodemographic, health status, and electronic device use were associated with Web 2.0 use for health information, data were examined for multicollinearity. Both the variance inflation factors (VIF) (≤1.23) and tolerance statistics (≤0.79) met their respective cut-off points of less than 10 and greater than 0.10 [57] indicating that the independent variables could reasonably be entered into multivariable analyses. In the multiple logistic regression analysis, the predictor variables were able to distinguish between use and non-use of social media for health information (χ^2^_19,283_=51.47, *P*=.001) by explaining a significant amount of variance in the model (Nagelkerke *R* ^*2*^ =.26). Table 4 lists the logistic regression coefficients for each predictor variable with associated 95% confidence intervals. Five of the predictor variables were significantly associated with use of Web 2.0 for health information: sex (*b*=0.97), possessing a baccalaureate (*b*=0.94) or post-graduate (*b*=1.96) degree, and self-reported use of one (*b*=1.30) or more than one (*b*=1.80) electronic device to find health information. Women were nearly three times more likely than men to use Web 2.0 for health information (OR 2.63, Wald= 8.09, df=1, *P*=.004), even after controlling for all other factors in the model. More education also predicted use of Web 2.0 for health information, with older college graduates over two times more likely than non-high school graduates to use Web 2.0 (OR 2.57, Wald= 3.86, df=1, *P*=.049). Respondents reporting a post graduate-level education were seven times more likely than non-high school graduates to use Web 2.0 for health information (OR 7.11, Wald=4.23, df=1, *P*=.04). In addition, when all other factors were held constant, respondents reporting use of one electronic device to search for health information were more than three times more likely to use Web 2.0 for health information than non-users of an electronic device for health information (OR 3.68, Wald=8.86, df=1, *P*=.003). Respondents reporting use of two or more electronic devices were more than six times more likely to report using Web 2.0 as compared to non-users (OR 6.06, Wald= 15.93, df=1, *P*=.001). Age, race, ethnicity, marital status, high school graduation, some college education, income, and health status did not significantly predict use of Web 2.0 for health information among respondents.

**Table 4:** Logistic regression predicting use of Web 2.0 for health information.

Sociodemographic variable		*B*	*SE B*	Exp (β)	95% CI
Constant		0.94	1.68	2.57	
Age		−0.03	0.02	0.98	0.95-1.02
Sex		0.97	0.34	2.63^b^	1.35-5.13
Ethnicity		0.03	0.66	1.03	0.29-3.75
Race		−0.28	0.17	0.77	0.56-1.08
**Marital status** ^c^					
	Widowed	−0.25	0.49	0.78	0.30-2.05
	Never married	−0.52	0.60	0.59	0.18-1.94
	Divorced or separated	−0.22	0.84	0.80	0.16-4.13
**Education** ^d^					
	High school graduate	0.33	0.59	1.39	0.44-4.39
	Some college	0.72	0.48	2.05	0.81-5.21
	4 years of college	0.94	0.48	2.57^a^	1.00-6.59
	Post graduate	1.96	0.95	7.11^a^	1.11-45.56
**Income (US$)** ^e^					
	$20,000 to $49,999	0.85	0.57	2.35	0.77-7.16
	$50,000 to $99,999	−0.40	0.47	0.67	0.27-1.69
	Over $100,000	−0.05	0.42	0.96	0.42-2.17
Health status		−0.20	0.15	0.82	0.61-1.09
Use of one electronic device for health information^f^		1.30	0.44	3.68^b^	1.56-8.68
Use of multiple electronic devices for health information^e^		1.80	0.45	6.06^b^	2.50-14.69

^a^ *P*<.05 two-tailed.

^b^ *P*<.01

^c^Reference category: Now married

^d^Reference category: Did not graduate high school

^e^Reference category: Less than $10,000

^f^Single electronic device use defined as self-reported use of 1 electronic device (ie, desktop, laptop, cell phone, tablet) to find Web-based health information.

^e^Multiple electronic device use defined as self-reported use of ≥2 electronic devices (ie, desktop, laptop, cell phone, tablet) to find Web-based health information.

## Discussion

### Principal Findings

Most sociodemographic variables (eg, gender, race/ethnicity, health status) and social determinants (eg, income, employment, marital status) examined in this study were not significant predictors of eHealth literacy or use of Web 2.0 for health information among baby boomers and older adults. However, education level, advanced age, and the extent to which electronic devices were used did appear to affect eHealth literacy. Level of education, electronic device use, and being female significantly influenced the use of Web 2.0 for health-related information.

### eHealth Literacy

The present study found that the majority of baby boomers and older adults used the Internet to find health information, and believed the Internet was useful for helping to make health decisions. While eHealth literacy scores decreased with age, they were comparable to scores reported in similar populations [12,31]. Overall, respondents in this study felt quite confident in their ability to use the Internet to find resources and answer questions about their health, yet they were less confident in their ability to evaluate Web-based health information. This finding is supported by Manafó and Wong [58], who reported that older adults lack confidence in their ability to discriminate between low- and high-quality health information. Research suggests that effective and user-friendly health promotion applications should be developed according to the intended audience’s eHealth literacy level [25]. Unfortunately, there are few eHealth literacy interventions that exist to increase user confidence among aging populations [58]. However, it is likely that baby boomers and older adults will display more confidence using eHealth tools over time, as individuals in these populations continue to adopt more technologically advanced digital devices [18]. Future research in the aging populations should focus on investigating how improved search functionality and e-communication skills may increase self-efficacy for finding age-appropriate, trustworthy health information on the Internet.

eHealth literacy was found to be influenced by age, education, and number of electronic devices used to search for health information in this study. Previous research notes that demographics, educational background, and technology use uniquely influences health literacy [28] and eHealth literacy [25] in the general population. Although having a higher level of education has been associated with more frequent use of the Internet for health information [12,59] and greater overall eHealth literacy in some instances [60], previous research indicates that more education is not always predictive of better eHealth literacy [12,55]. However, findings from this study suggest that baby boomers and older adults with more education have higher self-reported eHealth literacy. Because of the inconsistent findings regarding the association between education level and eHealth literacy in the aging population, further research is needed to further explore these relationships.

### Use of Web 2.0 for Health Information in Baby Boomers and Older Adults

Over one-third (35.7%) of respondents in this study indicated that they used some form of Web 2.0 to locate or share health information. This proportion is similar, yet slightly less, than the number reported in a 2010 Pew Research Center’s Internet and American Life Project survey, which found that 42% of Internet users over the age of 50 years had used Web-based social networking tools for general purposes in the past year [42]. Facebook and Twitter are Web 2.0 platforms most commonly used among individuals younger than 50 years old [61], yet the vast majority of older Web 2.0 users in this study reported use of these popular social networking technologies.

Internet and Web 2.0 users with a history of feeling socially isolated are less likely to perceive themselves as socially isolated when compared to individuals who do not utilize Internet or Web 2.0 [61,62]. The aging population may experience greater social support as a result of using Web 2.0 to connect with more powerful support networks [42]. The versatile elements of social media (eg, games, chat, shopping, health information) may provide these populations with more socially supportive venues to learn about their own health conditions and communicate with others who may be going through similar experiences. Future research should continue to explore the specific purposes that baby boomers and older adults have for using Web 2.0 to answer their health-related questions and/or communicate with others about their chronic health conditions.

Sex, education, and use of electronic devices to seek out health information significantly predicted use of Web 2.0 for health-related purposes among this random sample of baby boomers and older adults. Women were almost three times more likely to use Web 2.0 for health information than men. However, sex-related differences in the use of Web 2.0 for health information have been inconsistent in the general adult population. Chou and colleagues [37] found sex was not associated with social media use among US adults aged 18 years and older, and Elkin [63] noted that men were actually more likely than women to use social media to research health and wellness issues in a sample of adults between the ages of 18 and 80 years. Results from another national survey of US adults 18 years and older, indicated that women use social media to find health information 22% more often than men [64]. Some researchers have suggested that women are the primary health information seekers not only for themselves but also for loved ones, which may motivate their drive to find health information on the Internet [65]. Additional research is needed to clarify the precise role that sex plays in use of Web 2.0 for seeking and sharing health information among baby boomers and older adults, including the design and evaluation of Web 2.0 applications that target gender-specific health and informal caregiving needs.

Interestingly, age was not a significant predictor of utilizing Web 2.0 for health information, although it was a significant predictor of eHealth literacy. Kontos and colleagues found younger age to be the “primary driving factor” of social networking use among US adults, with use of social networking decreasing with age [66]. Although use of Web 2.0 for health information may decline with age, findings from this study suggest that the use of Web 2.0 for health information may bridge some generational gaps that extend beyond the baby boomer generation. Norman and Skinner suggest that the “more an individual uses technology, the more likely they are to develop skills in using that technology as a tool” [54]. Some researchers speculate that the phenomenon known as “the graying of social networking sites” may provide enumerable opportunities for providing health information to baby boomers and older adults in need of resources for health promotion and disease prevention [67].

In this study, race and ethnicity were not statistically significant predictors of Web 2.0 use for health information. Large, cross-sectional surveys have noted that Caucasians/whites, African Americans, and Latinos who use the Internet are all equally likely to use social networking applications for health-related purposes [68]. Kontos et al reported greater social networking use among racial/ethnic minorities and those with lower education and income levels [66]. Also, Chou and colleagues reported that African American Internet users in the United States are actually more likely than Caucasian Internet users to use social media for health communication [37]. While race and ethnicity were not significant predictors of Web 2.0 use for health information in the current study, these racial and ethnic minority groups were grossly underrepresented in the sample. Future research should investigate the use of Web 2.0 for health information among aging populations with diverse racial and ethnic backgrounds.

### Use of Web 2.0 for Health Information and eHealth Literacy

Respondents who used popular Web 2.0 websites (eg, Facebook, Twitter) and Web-based support groups for health-related purposes reported higher eHealth literacy than those who did not. While access to the Internet does not guarantee that individuals will be able to find, understand, evaluate, and act on Web-based health information [4,69], data from this study suggests that baby boomers and older adults who reported use of Web 2.0 for health-related purposes perceived themselves to have higher eHealth literacy [31]. LeRouge and colleagues report that barriers associated with utilizing technology among baby boomers and older adults are specific to the type of technology or device being used [46]. For example, baby boomers and older adults believe the mobile phone is an appropriate health-information technology, but they need more training to use it effectively for health-related purposes. While eHealth literacy has been described as a “learning system” of six discrete types of literacy that is not amenable to division [69], an updated definition of “eHealth literacy 2.0” is needed to account for the evolution of technology and the participative, social context of Web-based health information [24]. Computer (digital) and media literacies may actually be larger “petals” of eHealth literacy for baby boomer and older adult populations who need training and support to benefit from eHealth innovations. It is possible that baby boomers and older adults who learn to utilize Web 2.0 to locate and evaluate health information may gain Web-based social experiences that translate into better computer and media literacy skills. Therefore, to improve the ability of baby boomers and older adults to effectively access and utilize Web 2.0 for health care purposes, theory-based eHealth literacy interventions that apply high-quality research designs (eg, randomized controlled trials) should be evaluated in the aging population, particularly to measure effects on media and computer (digital) literacy related to health [70].

### Limitations

The current study possessed several limitations. The cross-sectional research design limits the researchers from establishing causation when considering the interrelationships between sociodemographic variables, social determinants, and health communication outcomes. In addition, the use of self-reported telephone surveys may have led participants to provide socially desirable responses [52]. For example, the interviewer was unable to provide respondents with visual cues or written definitions of potentially unfamiliar technical terms such as social media. Furthermore, the types of questions asked were somewhat restricted in scope, which resulted in data that was less rich than if in-person interviews were conducted with more exploratory, open-ended questions. The absence of visual and social cues may have also resulted in the loss of contextual and nonverbal data (eg, body language), which could have compromised responses and response interpretation [71]. Use of a follow-up Web survey would allow users to view the definitions of the social media tools discussed (eg, blogs) and likely decreased the demand on individuals’ cognitive burden [72]. Unlike during in-person interviews, the interviewer cannot see the individual to gauge their understanding of an item, and therefore may not provide clarification when it is needed [73]. It would be valuable to conduct an in-depth qualitative study of older adults who access Web-based health information using Web 2.0 tools.

Another limitation of this cross-sectional study was a lack of survey items that measured frequency of Internet and Web 2.0 use for health information. Previous research noted that more frequent access to computers and the Internet was associated with higher eHealth literacy [12,31] and more positive health behavior change [74]. Among the aging population, it is possible that frequency of Internet use and type of Internet access could affect both eHealth literacy and the use of Web 2.0 for health information. In future research, it will be important to explore the perceptions of older adults who access different types of Web 2.0 with variable frequency. Baby boomers and older adults who use popular social media may consider themselves to be simply involved in informal Web-based support groups (ie, they might not consider their affiliation with social media group to be official in nature). Among aging populations, it is unclear whether frequency of interaction and engagement (like, dislikes, comments, etc) on Web 2.0 is truly an active ingredient causing greater eHealth literacy. It may be that simple membership on social media sites/pages devoted to health might improve perceived knowledge and skills related to eHealth literacy.

Additionally, the eHEALS instrument is based on an individual’s perception of personal knowledge and skills related to eHealth literacy [75] rather than demonstrated eHealth literacy competencies. While the eHEALS is a valuable instrument for assessing Web 1.0 skills, it is unclear how accurately it measures use of Web 2.0 technologies to find and evaluate health information [24]. When eHEALS was developed, social media was still in its infancy; therefore, in this study, items assessing the use of social media for health information were adapted from HINTS [53]. It should be noted that eHealth literacy is an evolving concept that requires greater inquiry [76,77], and there may be a need for a more comprehensive survey instrument that assesses health information seeking and sharing using all types of Internet applications. This type of instrument should be culturally sensitive enough to administer in diverse populations, and may focus on types of Web-based health information sought, perceived goals of Web-based health information searching, and the use of different social media tools to communicate with others about health [78]. To date, an instrument of this kind has yet to be developed and validated.

One final limitation of this study was related to the landline sampling method that was employed, which excluded over one-third of the state population that owns only a mobile phone [79]. Individuals with mobile phones are more likely to be eHealth literate with greater social media savvy [80]. The landline sampling method could have also led to selection bias, as evidenced by the lower than expected minority representation in this study. Alternative sampling methods than the ones employed in this study may be needed to reach underrepresented populations such as Hispanic adults who are more likely than non-Hispanic white adults to be living in households with only wireless telephones [81].

### Conclusions

Web 2.0 has become a leading health communication platform and will continue to attract adult users of all ages; thus, it is important to continue to understand the impact of Web 2.0 on health information seeking and sharing among baby boomers and older adults. By 2015, use of the mobile Internet is projected to overtake conventional broadband Internet accessed through desktop computers [82]. As mobile apps continue to rapidly transform health care for seniors [67,83], future research should examine how mobile apps are being accessed among aging populations. The cross-sectional data from this study provides important new insights into select sociodemographics and social determinants that are associated with eHealth literacy levels and Web 2.0 use for health information in these populations. Specially, lower age and more education predicted higher eHealth literacy, and more education and being of female gender was associated with greater use of Web 2.0 for health information. Future interventions should consider providing access to tailored training opportunities based on age, education level, and gender, to improve use of advanced electronic devices to access Web-based health information. More in-depth qualitative studies with older populations are needed to better understand how and why aging populations use the Internet and Web 2.0 applications to locate and evaluate health information to make health-related decisions and solve health-related problems.

## Abbreviations

BEBR: University of Florida Bureau of Economic and Business Research
eHEALS: eHealth Literacy Scale
F-CCI: Florida Consumer Confidence Index
HINTS: Health Information National Trends Survey
OR: odds ratio
RDD: random digit dialing
SIMHC: Structural Influence Model of Health Communication
SPSS: Statistical Package for the Social Sciences
VIF: variance inflation factors

## References

[1] Internet World Stats Internet users in the world distribution by world regions – Q4 2014.

[2] Pew Internet & American Life Project Health Fact Sheet.

[3] Cline RJ, Haynes KM (2001). Consumer health information seeking on the Internet: the state of the art. Health Educ Res.

[4] Knapp Caprice, Madden Vanessa, Marcu Mircea, Wang Hua, Curtis Charlotte, Sloyer Phyllis, Shenkman Elizabeth (2011). Information seeking behaviors of parents whose children have life-threatening illnesses. Pediatr Blood Cancer.

[5] Muñoz Ricardo F (2010). Using evidence-based internet interventions to reduce health disparities worldwide. J Med Internet Res.

[6] Bodie Graham D, Dutta Mohan Jyoti (2008). Understanding health literacy for strategic health marketing: eHealth literacy, health disparities, and the digital divide. Health Mark Q.

[7] Bonnar-Kidd Kelly K, Black David R, Mattson Marifran, Coster Dan (2009). Online physical activity information: will typical users find quality information?. Health Commun.

[8] Connolly Kathleen Kihmm, Crosby Martha E (2014). Examining e-Health literacy and the digital divide in an underserved population in Hawai'i. Hawaii J Med Public Health.

[9] Hargittai E (2010). Digital Na(t)ives? Variation in internet skills and uses among members of the “Net Generation”. Sociological Inquiry.

[10] Lorence Daniel, Park Heeyoung (2008). Group disparities and health information: a study of online access for the underserved. Health Informatics J.

[11] Comerci GD (1988). Eating disorders in adolescents. Pediatr Rev.

[12] Neter Efrat, Brainin Esther (2012). eHealth literacy: extending the digital divide to the realm of health information. J Med Internet Res.

[13] Zajac Ian T, Flight Ingrid HK, Wilson Carlene, Turnball Deborah, Cole Steve, Young Graeme (2012). Internet usage and openness to internet-delivered health information among Australian adults aged over 50 years. Australas Med J.

[14] US Food and Drug Administration (2013). How to evaluate health information on the internet.

[15] Centers for Disease Control and Prevention (2011). Healthy aging.

[16] World Health Organization (2013). Health literacy: the solid facts.

[17] Sudore Rebecca L, Mehta Kala M, Simonsick Eleanor M, Harris Tamara B, Newman Anne B, Satterfield Suzanne, Rosano Caterina, Rooks Ronica N, Rubin Susan M, Ayonayon Hilsa N, Yaffe Kristine (2006). Limited literacy in older people and disparities in health and healthcare access. J Am Geriatr Soc.

[18] Smith A (2014). Older adults and technology use.

[19] (2014). Internet user demographics.

[20] Mobile technology fact sheet.

[21] Wagner N, Hassanein K, Head M (2010). Computer use by older adults: A multi-disciplinary review. Computers in Human Behavior.

[22] Medlock Stephanie, Eslami Saeid, Askari Marjan, Arts Derk L, Sent Danielle, de Rooij Sophia E, Abu-Hanna Ameen (2015). Health information-seeking behavior of seniors who use the Internet: a survey. J Med Internet Res.

[23] White Robert E (2008). Health information technology will shift the medical care paradigm. J Gen Intern Med.

[24] Norman Cameron (2011). eHealth literacy 2.0: problems and opportunities with an evolving concept. J Med Internet Res.

[25] Norman Cameron D, Skinner Harvey A (2006). eHealth Literacy: Essential skills for consumer health in a networked world. J Med Internet Res.

[26] Xie Bo (2011). Effects of an eHealth literacy intervention for older adults. J Med Internet Res.

[27] Weinstein Ronald S, Lopez Ana Maria (2014). Health literacy and connected health. Health Aff (Millwood).

[28] Institute of Medicine (2004). Health literacy: A prescription to end confusion.

[29] Zamora Herlinda, Clingerman Evelyn M (2011). Health literacy among older adults: a systematic literature review. J Gerontol Nurs.

[30] Xavier André J, d'Orsi Eleonora, de Oliveira Cesar M, Orrell Martin, Demakakos Panayotes, Biddulph Jane P, Marmot Michael G (2014). English Longitudinal Study of Aging: can Internet/E-mail use reduce cognitive decline?. J Gerontol A Biol Sci Med Sci.

[31] Choi Namkee G, Dinitto Diana M (2013). The digital divide among low-income homebound older adults: Internet use patterns, eHealth literacy, and attitudes toward computer/Internet use. J Med Internet Res.

[32] Xie B (2008). Lifelong Interactions: Older adults, health information, and the Internet. ACM Interactions.

[33] Aghaei S (2012). Evolution of the world wide web: from Web 1.0 to Web 4.0. IJWesT.

[34] Eysenbach Gunther (2008). Medicine 2.0: social networking, collaboration, participation, apomediation, and openness. J Med Internet Res.

[35] Gibbons M Chris, Fleisher Linda, Slamon Rachel E, Bass Sarah, Kandadai Venk, Beck J Robert (2011). Exploring the potential of Web 2.0 to address health disparities. J Health Commun.

[36] Fox S, Jones S (2009). The social life of health information.

[37] Chou Wen-ying Sylvia, Hunt Yvonne M, Beckjord Ellen Burke, Moser Richard P, Hesse Bradford W (2009). Social media use in the United States: implications for health communication. J Med Internet Res.

[38] Hardt JH, Hollis-Sawyer L (2007). Older adults seeking healthcare information on the Internet. Educational Gerontology.

[39] Lee Kenneth, Hoti Kreshnik, Hughes Jeffery David, Emmerton Lynne (2014). Dr Google and the consumer: a qualitative study exploring the navigational needs and online health information-seeking behaviors of consumers with chronic health conditions. J Med Internet Res.

[40] Cutrona SL, Roblin DW, Wagner JL, Gaglio B, Williams AE, Torres Stone R, Field TS, Mazor KM (2013). Adult willingness to use email and social media for peer-to-peer cancer screening communication: Quantitative interview study. JMIR Res Protoc.

[41] Capel Sue, Childs Sue, Banwell Linda, Heaford Susan (2007). Access to information and support for health: some potential issues and solutions for an ageing population. Health Informatics J.

[42] Madden M (2010). Older adults and social media.

[43] Stellefson Michael, Chaney Beth, Barry Adam E, Chavarria Enmanuel, Tennant Bethany, Walsh-Childers Kim, Sriram PS, Zagora Justin (2013). Web 2.0 chronic disease self-management for older adults: a systematic review. J Med Internet Res.

[44] Thackeray R, Crookston BT, West H (2013). Correlates of health-related social media use among adults. J Med Internet Res.

[45] Gatto SL, Tak SH (2008). Computer, internet, and e-mail use among older adults: benefits and barriers. Educational Gerontology.

[46] LeRouge Cynthia, Van Slyke Craig, Seale Deborah, Wright Kevin (2014). Baby boomers' adoption of consumer health technologies: survey on readiness and barriers. J Med Internet Res.

[47] Ackerson Leland K, Viswanath K (2009). The social context of interpersonal communication and health. J Health Commun.

[48] Viswanath K, Ramanadhan SR, Galea S, Kontos EZ (2007). Mass media. Macrosocial Determinants of Population Health.

[49] Colby SL, Ortman JM (2014). The baby boom cohort in the United States: 2012-2060.

[50] Coughlin J, D’Ambrosio LA, Reimer B, Pratt MR (2007). Older adult perceptions of smart home technologies: implications for research, policy & market innovations in healthcare. Conference Proceedings IEEE Engineering in Medicine and Biology Society.

[51] Bureau of Economic and Business Research.

[52] Dillman DA (2007). Mail and internet surveys: the tailored design method.

[53] Health Information National Trends Survey (2012). HINTS Questions: Internet use.

[54] Norman Cameron D, Skinner Harvey A (2006). eHEALS: The eHealth Literacy Scale. J Med Internet Res.

[55] van der Vaart Rosalie, van Deursen Alexander Jam, Drossaert Constance Hc, Taal Erik, van Dijk Jan Amg, van de Laar Mart Afj (2011). Does the eHealth Literacy Scale (eHEALS) measure what it intends to measure? Validation of a Dutch version of the eHEALS in two adult populations. J Med Internet Res.

[56] Korda Holly, Itani Zena (2013). Harnessing social media for health promotion and behavior change. Health Promot Pract.

[57] Pallant J (2010). SPSS Survival Manual: A step by step guide to data analysis using SPSS.

[58] Manafò E, Wong S (2012). Assessing the eHealth literacy skills of older adults: A preliminary study. Journal of Consumer Health On the Internet.

[59] Powell John, Inglis Nadia, Ronnie Jennifer, Large Shirley (2011). The characteristics and motivations of online health information seekers: cross-sectional survey and qualitative interview study. J Med Internet Res.

[60] van der Vaart Rosalie, Drossaert Constance HC, de Heus Miriam, Taal Erik, van de Laar Mart AFJ (2013). Measuring actual eHealth literacy among patients with rheumatic diseases: a qualitative analysis of problems encountered using Health 1.0 and Health 2.0 applications. J Med Internet Res.

[61] O’Brien C (2012). Participation in online communities and psychosocial well-being among older adults.

[62] (2014). Social networking fact sheet.

[63] Elkin N (2008). How America searches: health and wellness.

[64] Duggan M, Brenner J (2012). The demographics of social media users – 2013.

[65] Baur Cynthia (2008). An analysis of factors underlying e-health disparities. Camb Q Healthc Ethics.

[66] Kontos Emily Z, Emmons Karen M, Puleo Elaine, Viswanath K (2010). Communication inequalities and public health implications of adult social networking site use in the United States. J Health Commun.

[67] Boulos MNK (2012). Using social media for improving health literacy.

[68] Jones S, Fox S (2009). Generations online in 2009.

[69] Stellefson Michael, Hanik Bruce, Chaney Beth, Chaney Don, Tennant Bethany, Chavarria Enmanuel Antonio (2011). eHealth literacy among college students: a systematic review with implications for eHealth education. J Med Internet Res.

[70] Watkins Ivan, Xie Bo (2014). eHealth literacy interventions for older adults: a systematic review of the literature. J Med Internet Res.

[71] Novick Gina (2008). Is there a bias against telephone interviews in qualitative research?. Res Nurs Health.

[72] Dillman DA, Smyth JD, Christian LM (2014). Internet, phone, mail, and mixed-mode surveys: the tailored design method, 4th edition.

[73] Check J, Schutt R (2011). Research Methods in Education.

[74] Ayers Stephanie L, Kronenfeld Jennie Jacobs (2007). Chronic illness and health-seeking information on the Internet. Health (London).

[75] van Deursen Alexander JAM, van Dijk Jan AGM (2011). Internet skills performance tests: are people ready for eHealth?. J Med Internet Res.

[76] Chan Connie V, Kaufman David R (2011). A framework for characterizing eHealth literacy demands and barriers. J Med Internet Res.

[77] van Deursen A, van Dijk J, Peters O (2011). Rethinking internet skills: The contribution of gender, age, education, internet experience, and hours online to medium- and content-related internet skills. Poetics.

[78] Miller Lisa M, Bell Robert A (2012). Online health information seeking: the influence of age, information trustworthiness, and search challenges. J Aging Health.

[79] Blumberg SJ, Luke JV, Ganesh N, Davern ME, Bouderaux MH (2012). Natl Health Stat Report.

[80] Drury G (2008). Opinion piece: Social media: Should marketers engage and how can it be done effectively?. J Direct Data Digit Mark Pract.

[81] Stellefson Michael, Hanik Bruce, Chaney Beth, Chaney Don, Tennant Bethany, Chavarria Enmanuel Antonio (2011). eHealth literacy among college students: a systematic review with implications for eHealth education. J Med Internet Res.

[82] Charlton G (2012). Will mobile internet replace desktop?.

[83] Madden M, Zickuhr K (2011). 65% of online adults use social networking sites.

